# The impact of macrophage infiltration on [^18^F]FDG PET accuracy in identifying mediastinal and abdominal lymph node metastases: A retrospective cohort study

**DOI:** 10.1371/journal.pone.0340327

**Published:** 2026-01-23

**Authors:** Jing Wei, Weijing Tao, Tianshuo Yang, Jie Dong

**Affiliations:** 1 Department of Nuclear Medicine, The Affiliated Huai’an No.1 People’s Hospital of Nanjing Medical University, Huai’an, Jiangsu, China; 2 ECT Department, Huai’an Hospital, Huai’an, Jiangsu, China; Fondazione Policlinico Universitario Agostino Gemelli IRCCS, ITALY

## Abstract

This study aimed to comprehensively analyze the complex interaction between the presence of macrophages and [^18^F]FDG PET findings. This retrospective study enrolled 240 patients with histologically confirmed malignancies. Patients underwent histopathological examinations of excised lymph nodes, with CD163 staining to quantify M2 macrophage infiltration, which was used to stratify patients into high and low infiltration groups. Data collection encompassed demographic, clinical, imaging, and histological data. High infiltration group had a longer disease duration compared to the low infiltration group (p < 0.001). [^18^F]FDG PET imaging showed that the high infiltration group had significantly higher median SUVmax values compared to the low infiltration group (p < 0.001), and a higher incidence of lymph node metastasis (p = 0.039). Sensitivity and specificity of [^18^F]FDG PET imaging were 0.795 and 0.619 for the high infiltration group, and 0.582 and 0.785 for the low infiltration group, respectively. ROC analysis demonstrated a higher diagnostic accuracy in the high infiltration group (AUC 0.784) compared to the low infiltration group (AUC 0.737). A strong positive correlation was observed between macrophage infiltration levels and PET SUVmax values (p < 0.001), and a significant correlation was also noted between macrophage infiltration and the presence of lymph node metastasis (p = 0.003). In conclusion, increased CD163+M2 macrophage infiltration correlates with higher SUVmax values and more frequent detection of lymph node metastases. These findings suggest that macrophage infiltration may influence [^18^F]FDG PET imaging performance, potentially contributing to variations in diagnostic accuracy.

## Introduction

In the clinical management of cancer, lymph node metastasis is a critical issue that involves the spread of cancer cells from the primary tumor site through the lymphatic system to different lymph nodes in the body. For instance, in breast and lung cancers, the presence of lymph node metastasis is a key factor distinguishing early-stage from advanced-stage cancer [1,2]. Common diagnostic modalities for lymph node evaluation include ultrasound, computed tomography (CT), magnetic resonance imaging (MRI), and positron emission tomography (PET). Among these, PET scanning is particularly noted for its high sensitivity in assessing lymph node involvement by tumors [3]. Studies have demonstrated that 2-deoxy-2-[^18^F]fluoro-D-glucose ([^18^F]fluorodeoxyglucose, [^18^F]FDG) PET/CT imaging has a high negative predictive value in detecting cervical lymph node metastasis in patients with newly diagnosed, untreated oral squamous cell carcinoma [4]. However, Hope et al. found that while [^18^F]FDG PET has high specificity, its sensitivity for diagnosing pelvic lymph nodes in high-risk prostate cancer patients is relatively low [5]. Additionally, challenges remain in the identification of mediastinal and abdominal lymph node metastases using [^18^F]FDG PET, including the oversimplification of the tumor microenvironment and the underestimation of the role of immune cells [6–8].

Macrophages, as part of the mononuclear phagocyte system, are critical cells within the immune system. Their primary functions include clearing cellular debris, combating infections, and regulating immune responses. Increasing evidence suggests that macrophages within the tumor microenvironment are closely associated with tumor progression and prognosis [9]. Macrophages not only promote tumor proliferation and invasion but also play a significant role in tumor immunotherapy, especially CD163, a molecule that specifically marks macrophages, especially M2 macrophages [10,11]. Previous studies have found that CD163 expression in cancer cells is positively correlated with macrophage infiltration and strong macrophage infiltration is associated with better cancer-specific survival, but macrophage infiltration is negatively correlated with lymph node metastasis [12,13]. High levels of M2 macrophage infiltration are generally linked to poor prognosis, a finding validated in both bladder cancer [12] and breast cancer [13]. There is also a significant correlation between CD163+ M2 macrophages and lymph node metastasis of colorectal cancer [14]. However, it has been observed that the presence and activity of macrophages may lead to false-positive results in [^18^F]FDG PET imaging, thereby affecting the accuracy of tumor metastasis assessment [8].

Therefore, this study aims to investigate the potential impact of macrophage infiltration on [^18^F]FDG PET imaging in detecting mediastinal and abdominal lymph node metastasis through systematic [^18^F]FDG PET image analysis. By thoroughly examining the relationship between macrophages and [^18^F]FDG PET imaging, we hope to uncover their role in tumor diagnosis, providing more reliable evidence for clinical decision-making. This research is expected to offer new insights into personalized treatment and the advancement of precision medicine.

## Methods

### Participants

**Fig 1:**
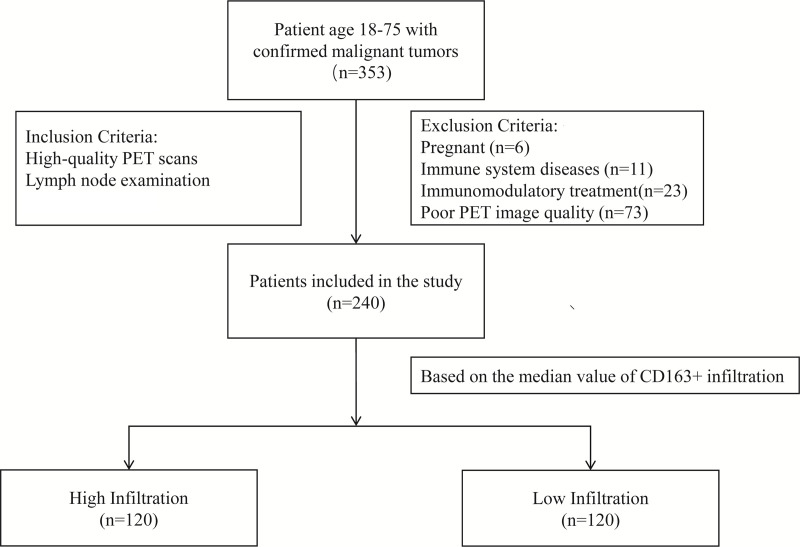
Study design and patient selection flowchart.

This retrospective study recruited patients from our hospital between 31/01/2021 to 31/12/2023. The patient data were accessed for research purposes between 31/01/2024 to 15/02/2024. Eligible participants were adults aged 18–75 years. The inclusion criteria were a confirmed diagnosis of a malignant tumor, the requirement for lymph node assessment, and high-quality [^18^F]FDG PET scans available for analysis. Exclusion criteria included pregnancy, known immune system diseases, ongoing immunomodulatory treatment, and poor quality of [^18^F]FDG PET images that could impair diagnostic accuracy. A total of 240 patients met the inclusion criteria and were enrolled in the study from an initial pool of 353 assessed for eligibility (Fig 1). This study was approved by the Institutional Review Board of The Affiliated Huai’an No.1 People’s Hospital of Nanjing Medical University. All procedures involving human participants adhered to the ethical standards of the Declaration of Helsinki and its subsequent amendments. Informed consent was waived by the Institutional Review Board of The Affiliated Huai’an No.1 People’s Hospital of Nanjing Medical University due to the retrospective nature of the study.

### [^18^F]FDG PET imaging

[^18^F]FDG PET scans were performed using a Siemens Biograph PET/CT system (syngo MI.PET/CT 2012A; Siemens Healthineers, Erlangen, Germany). Before imaging, patients fasted for at least 6 hours and had a blood glucose level below 9.0 mmol/L. A dose of [^18^F]FDG with radiochemical purity >95% was administered intravenously via the antecubital vein at 3.70–5.55 MBq/kg. After injection, patients rested 60 min in the supine position and emptied the bladder immediately before scanning. The acquisition covered skull vertex to proximal femora. CT-based attenuation correction was applied, and PET data were reconstructed with an iterative algorithm, then fused with CT in axial, sagittal, and coronal planes. Two senior nuclear medicine physicians independently reviewed the images. Visually apparent [^18^F]FDG PET data were reconstructed with an iterative algorithm, and the maximum standard uptake values (SUVmax) were calculated. uptake higher than surrounding tissues and not corresponding to normal anatomic structures was considered PET-positive by consensus. Quantitative analysis used the vendor’s workstation and SUV_max_ was obtained for each lesion.

### Histopathological examination

For patients undergoing surgical management of their tumors, excised lymph nodes were subjected to histopathological examination. This involved staining with hematoxylin and eosin, followed by immunohistochemical staining for CD163 to quantify M2 macrophage infiltration. The median value of CD163+ cells per high power field was used to stratify patients into high and low macrophage infiltration groups.

### Data collection

Demographic and clinical data included the patient’s age, sex, tumor type, Disease Duration, and the pathological stage of the tumor. Imaging data were collected from [^18^F]FDG PET scans, with a special focus on the maximum standard uptake values (SUVmax). Histological data were obtained through immunohistochemical analysis to determine the level of CD163+ M2 macrophage infiltration in the lymph nodes.

### Statistical analysis

The data analysis was performed using SPSS 26.0. The normality of the distribution for continuous variables using the Shapiro-Wilk test. Variables that conformed to a normal distribution were described using means ± standard deviations (SD). In contrast, variables that did not exhibit normal distribution were presented using medians and interquartile ranges. Categorical data were summarized as counts and percentages. The independent samples t test or Mann-Whitney U test was used to compare continuous variables between the two groups, and the χ² test or Fisher’s exact test was used for categorical variables. The PET positivity threshold was prespecified from the overall cohort by maximizing Youden’s index for discriminating pathologically proven nodal metastasis based on SUV_max_. In addition, Pearson correlation analysis was used to evaluate the correlation between the degree of macrophage infiltration and [^18^F]FDG PET image features. Receiver Operating Characteristic (ROC) curves was performed to evaluate the accuracy of [^18^F]FDG PET in identifying mediastinal and abdominal lymph node metastases. All tests were two-tailed, and a p-value of less than 0.05 was considered statistically significant.

## Result

### General characteristics

The cohort was stratified into two groups based on the median value of CD163+ M2 macrophage infiltration, which was 107: a high macrophage infiltration group (n = 120) and a low macrophage infiltration group (n = 120). The demographic and clinical characteristics of the participants were balanced between the two groups, with no significant differences in age, sex, or tumor type, ensuring comparability. Both groups had a median age of 48 years, with a nearly equal male-to-female ratio. However, a significant difference was noted in the disease duration, with the high macrophage infiltration group presenting a longer mean duration of 16.60 months compared to 10.15 months in the low infiltration group (P < 0.001) (Table 1).

**Table 1:** Characteristics of patients with high and low macrophage infiltration.

Parameter	High Macrophage Infiltration (n = 120)	Low Macrophage Infiltration (n = 120)	P value
**Age (years)**	48.00 (34.00, 61.25)	48.00 (32.00, 62.00)	0.770
**Sex (Male)**	57 (47.50%)	59 (49.17%)	0.897
**Tumor Type**			0.789
Lung	42 (35.00%)	37 (30.83%)	
Colorectal	42 (35.00%)	45 (37.50%)	
Stomach	36 (30.00%)	38 (31.67%)	
**Disease Duration (months)**	16.60 (8.60, 24.00)	10.15 (6.10, 23.78)	0.029*
**Pathological Stage**			0.453
Stage I	21 (17.50%)	27 (22.50%)	
Stage II	47 (39.17%)	50 (41.67%)	
Stage III	52 (43.33%)	43 (35.83%)	

**Notes**: Data are presented as median (25th percentile, 75th percentile) for non-normally distributed variables. Sex, Tumor Type and Pathological Stage are presented as count (percentage). P-value < 0.05 is considered statistically significant and is marked with an asterisk (*).

### [^18^F]FDG PET imaging and histopathological findings

The [^18^F]FDG PET imaging results revealed a statistically significant difference in SUVmax measured from lymph nodes between high and low macrophage infiltration groups, and also between long and short disease duration groups.

The high macrophage infiltration group had a median SUVmax of 10.20, which was significantly higher than the 5.82 observed in the low macrophage infiltration group (Table 2, p < 0.001). Correspondingly, a higher incidence of lymph node metastasis was noted in the high infiltration group, with 59% (71/120) testing positive compared to 45.83% (55/120) in the low infiltration group (Table 2, p = 0.039). Across all three cancers, nodes in the CD163-high group showed higher SUVmax (all P < 0.001), and the metastasis rate was higher in lung and colorectal but not in stomach cancer (S1–S3 Tables). Notably, SUVmax was consistently higher in the high macrophage infiltration group compared to the low infiltration group, both in metastatic and non-metastatic lymph nodes (S4 Table).

**Table 2:** PET results and histopathological findings by degree of macrophage infiltration.

Parameter	High Macrophage Infiltration (n = 120)	Low Macrophage Infiltration (n = 120)	P value
**PET SUVmax**	10.20 (8.75, 11.92)	5.82 (4.99, 6.71)	<0.001*
**Lymph node metastasis (Positive)**	71 (59.17%)	55 (45.83%)	0.039*

**Notes**: Data are presented as median (25th percentile, 75th percentile) for non-normally distributed variables. Lymph Node Metastasis is presented as count (percentage). P-value < 0.05 is considered statistically significant and is marked with an asterisk (*).

We also stratified the cohort by the median value of disease duration, which was 14.15 months. The long disease duration group had a median SUVmax of 9.00 months, significantly higher than that of short disease duration group, which was 6.38 months (Table 3, p < 0.001). This aligns with the finding in lymph node metastasis, where 70.83% (85/120) of the patients was tested positive in the long disease duration group, while in the short disease duration group, the number was 34.17% (41/120) (Table 3, p < 0.001).

**Table 3:** PET results and histopathological findings by disease duration.

Parameter	Short Disease Duration (n = 120)	Long Disease Duration (n = 120)	P value
**PET SUVmax**	6.38 (5.22, 8.46)	9.00 (6.61, 11.32)	<0.001*
**Lymph Node Metastasis (Positive)**	41 (34.17%)	85 (70.83%)	<0.001*

### Sensitivity and specificity of [^18^F]FDG PET imaging

**Fig 2:**
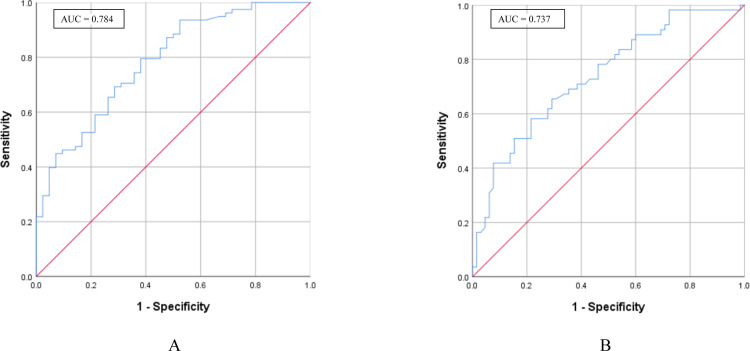
ROC curve analysis of PET accuracy in identifying lymph node metastases with different levels of macrophage infiltration. **(A)** ROC curve for PET accuracy in patients with high macrophage (CD163+ cells) infiltration. The AUC value is 0.784. **(B)** ROC curve for PET accuracy in patients with low macrophage (CD163+ cells) infiltration. The AUC value is 0.737.

Evaluations of the sensitivity and specificity of [^18^F]FDG PET imaging for detecting lymph node metastases showed distinct outcomes. The high infiltration group exhibited a sensitivity of 0.795 and a specificity of 0.619, contrasting with a sensitivity of 0.582 and a specificity of 0.785 in the low infiltration group (Table 4). The Area Under the Curve (AUC) from the ROC analysis indicated better diagnostic accuracy in the high infiltration group (0.784) compared to the low infiltration group (0.737) (Fig 2). Using the prespecified threshold (SUV_max_ ≥ 6.885), a total of 55 lymph nodes were classified as false positives. These false-positive findings occurred in both sexes (29 males and 26 females) and across a wide age range (19–74 years). With respect to primary tumor type, false positives were more frequently observed in colorectal cancer (n = 24), followed by gastric cancer (n = 19) and lung cancer (n = 12).

**Table 4:** Comparison of sensitivity and specificity of PET in identification of mediastinal and abdominal lymph node metastases.

Parameter	High Macrophage Infiltration (n = 120)	Low Macrophage Infiltration (n = 120)
**Sensitivity**	0.795	0.582
**Specificity**	0.619	0.785
**AUC (95%CI)**	0.784 (0.700–0.868)	0.737 (0.648–0.825)
**P value**	<0.001	<0.001

**Notes**: Sensitivity: AUC: Area Under the Curve, representing the diagnostic accuracy of PET SUVmax. P-value < 0.05 is considered statistically significant and is marked with an asterisk (*).

### Correlation analysis

A moderate positive correlation was found between the level of macrophage infiltration and PET SUVmax values (correlation coefficient = 0.729, p < 0.001). Additionally, the correlation between macrophage infiltration and the presence of lymph node metastasis was weak, but statistically significant (correlation coefficient = 0.134, p = 0.039) (Table 5).

**Table 5:** Correlation analysis between macrophage infiltration and PET characteristics.

Parameter	Correlation Coefficient	P value
**PET SUVmax**	0.729	<0.001*
**Lymph Node Metastasis**	0.134	0.039*

**Notes**: Correlation Coefficient is calculated using Pearson correlation for continuous variables and point-biserial correlation for binary variables. P-value < 0.05 is considered statistically significant and is marked with an asterisk (*).

## Discussion

Tumor metastasis is one of the primary causes of poor prognosis in cancer patients, with mediastinal and abdominal lymph node metastases being particularly critical for many types of cancer [15]. In this study, we investigated the impact of macrophage infiltration, specifically CD163+ M2 macrophages, on the diagnostic accuracy of [^18^F]FDG PET imaging in identifying mediastinal and abdominal lymph node metastases in cancer patients. CD163 was selected as the stratifying marker in this study due to its well-established role as a highly specific marker for M2-polarized tumor-associated macrophages, which play pivotal roles in immunosuppression, tumor progression, and metastatic dissemination [16]. For instance, a study by Chen et al. compared multiple immune-related markers (CD163, CD68, CD8, CD4) and found that although SUV_max_ correlated positively with all four, only CD163 expression remained independently associated with SUVmax in multivariate logistic regression analysis, along with histological tumor grade [17].

Our findings indicate that there is a complex interaction between the tumor microenvironment and imaging biomarkers. In our study, patients with higher levels of macrophage infiltration had longer disease durations compared to those with lower levels of infiltration, which is consistent with previous research [18]. Additionally, numerous studies have confirmed that high levels of M2 macrophage infiltration are associated with poorer chemotherapy response and overall survival [19,20]. Furthermore, our [^18^F]FDG PET image analysis revealed that patients with high macrophage infiltration exhibited significantly higher metabolic activity in their lymph node regions, with a correspondingly higher incidence of lymph node metastasis. This suggests a strong correlation between high SUVmax values and mediastinal lymph node metastasis, indicating that higher SUVmax values are associated with a greater likelihood of lymph node metastasis [16]. Usuda et al. evaluated the role of SUVmax in assessing the malignancy of mediastinal lymph nodes and found that patients with higher SUVmax values were more likely to have malignant lymph node metastasis [21]. Similarly, in patients with abdominal lymph node metastasis, the affected lymph node regions exhibited higher metabolic activity and SUVmax values [20].

In addition to macrophage infiltration, we also examined the role of disease duration in relation to PET imaging and lymph node metastasis. Stratification by the median disease duration (14.15 months) revealed that patients in the long-duration group had significantly higher SUVmax values and a markedly increased rate of lymph node metastasis compared to those in the short-duration group. These findings suggest that longer disease duration may be associated with greater tumor metabolic activity and more advanced disease spread. This is consistent with previous findings showing that longer disease duration may be associated with more advanced tumor features and lymph node metastasis in esophagogastric junction cancers [22].

Another notable observation of this study is that the presence of macrophage infiltration may be a crucial factor in enhancing the accuracy of [^18^F]FDG PET in identifying mediastinal and abdominal lymph node metastases. Our results indicate that, compared to the low macrophage infiltration group, the high macrophage infiltration group exhibited better [^18^F]FDG PET diagnostic performance. Although the high macrophage infiltration group demonstrated a higher AUC (0.784), indicating improved overall diagnostic accuracy, its specificity (0.619) was notably lower compared to the low infiltration group (0.785). This apparent discrepancy may be explained by the increased [^18^F]FDG uptake associated with high levels of macrophage activity. CD163+ M2 macrophages are metabolically active and can accumulate [^18^F]FDG even in the absence of malignancy, thereby increasing the rate of false-positive findings. Consequently, while sensitivity improves due to heightened tracer uptake in metastatic nodes, the trade-off is a reduced specificity caused by [^18^F]FDG -avid but benign inflammatory sites. This dynamic underscores that a higher AUC reflects improved overall discriminatory power, but not necessarily optimized performance at a fixed threshold of specificity.

This finding is in line with the results of Zarif et al., who found that in prostate cancer patients, lymph node regions with high CD163+ M2 macrophage infiltration showed higher metabolic activity (SUVmax) on PET/CT scans. This elevated metabolic activity helps improve the diagnostic accuracy of PET/CT for lymph node metastasis [19]. In bladder cancer, PET/CT also demonstrates high sensitivity and specificity in evaluating lymph node metastasis, particularly in the mediastinal and abdominal lymph node regions, where higher metabolic activity is closely associated with CD163+ M2 macrophage infiltration levels [23]. However, some studies have shown that the diagnostic efficacy of [^18^F]FDG PET may not significantly improve, or may even be affected, in cases of high macrophage infiltration. For instance, Lee et al. found that the sensitivity of [^18^F]FDG PET was lower than that of CT in detecting mediastinal lymph node metastasis, suggesting that [^18^F]FDG PET’s diagnostic performance may be limited by the degree of macrophage infiltration in certain situations [24]. Similarly, Atri et al. reported that while PET/CT improved sensitivity in detecting abdominal lymph node metastasis in high-risk endometrial cancer patients, it did not significantly outperform CT alone in terms of specificity and overall diagnostic accuracy [25]. Additionally, Yoon et al. found that although [^18^F]FDG PET was more sensitive than CT in detecting lymph node metastasis in esophageal squamous cell carcinoma, its specificity was lower, particularly in assessing mediastinal and abdominal lymph node metastasis, leading to a higher rate of false-positive results [26]. Looking forward, although the present study used static PET/CT with SUV-based analysis, there is growing evidence that dynamic [¹⁸F]FDG PET combined with compartmental kinetic modeling may significantly enhance diagnostic specificity by disentangling tracer delivery (K₁) from intracellular phosphorylation and retention (k₃), which are often conflated in SUV measurements [27,28]. This distinction is particularly critical in differentiating malignant lesions from inflammation-driven uptake in lymph nodes, where SUV-based assessments can lead to false positives [28,29]. We plan to evaluate dynamic approaches prospectively to test whether they add value beyond SUV_max_ in reducing inflammation-related false positives.

This study has several limitations. First, it is a retrospective, single-center analysis restricted to surgical cases, which may introduce selection bias and limit generalizability. Second, because SUV_max_ of primary and histological subtypes were not systematically recorded across all cases, precluding matching or uniform adjustment and leaving potential residual confounding. Additionally, individual biological differences could influence the outcomes, necessitating further validation in future research. Lastly, CD163 was used as a single macrophage marker and does not capture the full macrophage landscape, and PET evaluation focused on SUV_max_ without assessing other metabolic parameters such as metabolic tumor volume (MTV) and total lesion glycolysis (TLG). We plan to incorporate these parameters in future studies for a more comprehensive evaluation.

## Conclusion

In conclusion, our findings indicate that CD163+ M2 macrophage infiltration significantly affects the accuracy of [^18^F]FDG PET imaging in detecting mediastinal and abdominal lymph node metastasis. A deeper understanding of these dynamics could improve diagnostic protocols and potentially lead to the development of strategies that modulate the tumor microenvironment, thereby reducing the confounding impact of macrophages on imaging results.

## Supporting information

S1 Table PET results and histopathological findings by degree of macrophage infiltration in colorectal cancer. (DOCX)

S2 Table PET results and histopathological findings by degree of macrophage infiltration in lung cancer. (DOCX)

S3 Table PET results and histopathological findings by degree of macrophage infiltration in stomach cancer. (DOCX)

S4 Table PET results by degree of macrophage infiltration stratified by lymph node metastasis status. (DOCX)
