# Supplementary material for: The impact of macrophage infiltration on [18F]FDG PET accuracy in identifying mediastinal and abdominal lymph node metastases: A retrospective cohort study
Source: PLoS One. 2026 Jan 23;21(1):e0340327. doi: 10.1371/journal.pone.0340327 (PMC12829846; doi:10.1371/journal.pone.0340327)
Supplement: S1 Table — (DOCX) [file pone.0340327.s001.docx]

**Table S1 PET Results and Histopathological Findings by Degree of Macrophage Infiltration in Colorectal Cancer**

| **Parameter** | **High Macrophage Infiltration (n=42)** | **Low Macrophage Infiltration (n=45)** | **P value** |
| --- | --- | --- | --- |
| **PET SUVmax** | 10.35±2.20 | 5.96±1.21 | <0.001 |
| **Lymph Node Metastasis (Positive)** | 20 (47.62%) | 12 (26.67%) | 0.043 |
