# Supplementary material for: The impact of macrophage infiltration on [18F]FDG PET accuracy in identifying mediastinal and abdominal lymph node metastases: A retrospective cohort study
Source: PLoS One. 2026 Jan 23;21(1):e0340327. doi: 10.1371/journal.pone.0340327 (PMC12829846; doi:10.1371/journal.pone.0340327)
Supplement: S2 Table — (DOCX) [file pone.0340327.s002.docx]

**Table S2 PET Results and Histopathological Findings by Degree of Macrophage Infiltration in Lung Cancer**

| **Parameter** | **High Macrophage Infiltration (n=42)** | **Low Macrophage Infiltration (n=37)** | **P value** |
| --- | --- | --- | --- |
| **PET SUVmax** | 9.97±3.03 | 5.68±1.35 | <0.001 |
| **Lymph Node Metastasis (Positive)** | 31 (73.81%) | 18 (48.65%) | 0.021 |
