# Supplementary material for: The impact of macrophage infiltration on [18F]FDG PET accuracy in identifying mediastinal and abdominal lymph node metastases: A retrospective cohort study
Source: PLoS One. 2026 Jan 23;21(1):e0340327. doi: 10.1371/journal.pone.0340327 (PMC12829846; doi:10.1371/journal.pone.0340327)
Supplement: S3 Table — (DOCX) [file pone.0340327.s003.docx]

**Table S3 PET Results and Histopathological Findings by Degree of Macrophage Infiltration in Stomach Cancer**

| **Parameter** | **High Macrophage Infiltration (n=36)** | **Low Macrophage Infiltration (n=38)** | **P value** |
| --- | --- | --- | --- |
| **PET SUVmax** | 11.39±3.14 | 5.88±1.36 | <0.001 |
| **Lymph Node Metastasis (Positive)** | 20 (55.56%) | 25 (65.79%) | 0.367 |
