# Supplementary material for: The impact of macrophage infiltration on [18F]FDG PET accuracy in identifying mediastinal and abdominal lymph node metastases: A retrospective cohort study
Source: PLoS One. 2026 Jan 23;21(1):e0340327. doi: 10.1371/journal.pone.0340327 (PMC12829846; doi:10.1371/journal.pone.0340327)
Supplement: S4 Table — (DOCX) [file pone.0340327.s004.docx]

**Table S4 PET Results by Degree of Macrophage Infiltration** **Stratified by Lymph Node Metastasis Status**

| **Lymph Node Metastasis** | **Macrophage Infiltration** | **PET SUVmax** | **P value** |
| --- | --- | --- | --- |
| **Positive** | High (n=71) | 11.05±3.27 | <0.001 |
|  | Low (n=55) | 6.07±1.30 |  |
| **Negative** | High (n=49) | 9.77±1.85 | <0.001 |
|  | Low (n=65) | 5.65±1.27 |  |
